# Unidentified dengue serotypes in DENV positive samples and detection of other pathogens responsible for an acute febrile illness outbreak 2016 in Cajamarca, Peru

**DOI:** 10.1186/s13104-020-05318-5

**Published:** 2020-10-06

**Authors:** Juana del Valle-Mendoza, Fernando Vasquez-Achaya, Miguel Angel Aguilar-Luis, Johanna Martins-Luna, Jorge Bazán-Mayra, Victor Zavaleta-Gavidia, Wilmer Silva-Caso, Hugo Carrillo-Ng, Yordi Tarazona-Castro, Ronald Aquino-Ortega, Luis J. del Valle

**Affiliations:** 1 School of Medicine, Research and Innovation Center of the Faculty of Health Sciences, Universidad Peruana de Ciencias Aplicadas, Av. San Marcos cuadra 2, Chorrillos, Lima, Peru; 2 Laboratorio de Biologia Molecular, Instituto de Investigación Nutricional, Lima, Peru; 3 Laboratorio Regional de Cajamarca, Dirección Regional de Salud de Cajamarca (DIRESA), Cajamarca, Peru; 4 Escuela Profesional de Genética y Biotecnología. Facultad de Ciencias Biológicas, Universidad Nacional Mayor de San Marcos, Lima, Peru; 5 Barcelona Research Center for Multiscale Science and Engineering, Departament D’Enginyeria Química, EEBE, Universitat Politècnica de Catalunya (UPC), Barcelona, Spain

**Keywords:** Peru, Arbovirus, Dengue, Chikungunya, Zika, PCR

## Abstract

**Objective:**

To describe the prevalence of dengue virus serotypes, as well as other viral and bacterial pathogens that cause acute febrile illness during an outbreak in Cajamarca in 2016.

**Results:**

Dengue virus (DENV) was the most frequent etiologic agent detected in 25.8% of samples (32/124), followed by *Rickettsia *spp. in 8.1% (10/124), Zika virus in 4.8% (6/124), Chikungunya virus 2.4% (3/124) and *Bartonella bacilliformis* 1.6% (2/124) cases. No positive cases were detected of Oropouche virus and *Leptospira* spp. DENV serotypes identification was only achieved in 23% of the total positive for DENV, two samples for DENV-2 and four samples for DENV-4.

During the 2016 outbreak in Cajamarca—Peru, it was observed that in a large percentage of positive samples for DENV, the infecting serotype could not be determined by conventional detection assays. This represents a problem for the national surveillance system and for public health due to its epidemiological and clinical implications. Other viral and bacterial pathogens responsible for acute febrile syndrome were less frequently identified.

## Introduction

Fever is one of the most common symptoms reported by patients seeking medical attention in resource-limited settings [1]. Acute febrile illness (AFI) is a clinical syndrome that emcompasses a wide variety of differential diagnosis, which is caused by several infectious diseases that contribute to the global burden of morbidity and mortality [1, 2]. AFI can be caused by emerging and reemerging infectious diseases, such as viral and bacterial pathogens with high epidemic potential [3]. The study of these infections can reveal the evolutionary properties of their causative agents and the dynamic interaction with their hosts and environment [4, 5, 6].

In reference to viral pathogens, arthropod-borne viruses have reemerged in several geographic regions becoming a major health problem [6, 7, 8]. These viruses are in continuous expansion due to the effect of globalization, climate change, travel, vector expansion, among others [9]. In South America, the Dengue virus (DENV), whose reservoir and amplifying host are humans, is the most recognized arbovirus due to the co-circulation of 4 divergent serotypes with implications in the clinical presentation of the disease and viral epidemiology.[7, 8, 10, 11]. In Peru, the increasing prevalence of DENV has caused different outbreaks, reaching up to 35,000 confirmed cases per year, and largely affecting the southern region of Cajamarca [12]. However, in recent years other emerging viral pathogens have gained attention due to their increasing detection in Peru and Latin America, such as Zika, Chikungunya and Oropuche virus, with epidemiological implications still unknown in our population [4, 7, 13].

Regarding bacterial pathogens, 26 important emerging and reemerging bacterial infectious diseases have been described worldwide in the last 50 years; *Bartonella* and *Rickettsia* being among the most important causes of AFI [14]. Both pathogens are frequently reported in different regions of Peru, moreover studies have identified the presence of underported *Rickettsia* species in humans in four regions of the Peruvian territory [15, 16, 18].

Therefore, the main objective of this study was to determine the prevalence of dengue virus serotypes, as well as other possible etiological agents responsible for an acute febrile illness outbreak [14] in the region of Cajamarca.

## Main text

### Methods

#### Study location

A consecutive cross-section study was conducted in the Department of Cajamarca, Peru located in northern highlands of the country with a total population of 1 341 012. Cajamarca is an endemic area for Dengue serotypes 2 and 3 with outbreaks registering up to 3200 confirmed cases per year [12]. Moreover, other causes of AFI have been reported in this area such as Zika virus (ZIKV), Chikungunya virus (CHIKV), Oropouche virus (OROV), as well as bacterial pathogens including *Bartonella* spp, *Rickettsia* spp, and *Leptospira* spp. Nonetheless, malaria is not considered an endemic disease in the current study locaiton.

#### Patients and sampling

Serum samples from patients with acute febrile illness with suspicious of dengue infection were collected from February to June 2016 for etiological confirmation via PCR. The inclusion criteria were patients who presented to outpatient health centers with acute febrile illness, defined as an axillary temperature greater than or equal to 38 °C within at least 7 days prior to consultation without an identifiable source of infection. A suspected case of dengue was defined as per the CDC 2015 case definition. As part of the national surveillance system in Cajamarca, all suspected cases for dengue from all 12 provinces of Cajamarca are referred to a sentinel hospital for sample collection followed by serological confirmation by the National Institute of Health in Lima, Peru. Unfortunately, due to resources limitations, less than 50% can be laboratory-confirmed [4]. Our research team collected all blood samples from the healthcare center “Centro de Salud Chilete” for testing all the samples in our laboratory as an aid to our national surveillance system. All patient who fulfilled the selection criteria were included in the study for PCR detection of Dengue virus (DENV) serotypes, Zika virus (ZIKV), Chikungunya virus (CHIKV), Oropouche virus (OROV), as well as bacterial pathogens included in the differential diagnosis of AFI, including *Bartonella* spp, *Rickettsia* spp, and *Leptospira* spp.

#### Ethics statement

This study was approved by the Research Ethics Board of the *Hospital Regional de Cajamarca,* Peru. The samples were collected within the framework of the epidemiological surveillance program of acute febrile syndrome in the Cajamarca Region. According to international ethical guidelines for research related to human health prepared by CIOMS and WHO, ethics or informed consent is not required.

#### Samples

A total of 124 samples were collected by using Vacuette® TUBE Serum Separator Clot Activator (Vacuette, Greiner Bio-One, Kremsmünster, Austria). All the samples were stored at −80 °C after collection for molecular assays.

#### Molecular detection of OROV, DENV, CHIKV, and ZIKV

RNA extraction was performed from 200 μL of serum samples, RNA was extracted with the High Pure RNA Isolation Kit (Roche Applied Science, Mannheim, Germany), according to the manufacturer’s instructions.

Amplification by PCR assay for the detection of OROV was carried out using the primers described by Moreli et al. [14], and PCR conditions described by Silva-Caso et al. [9]. Amplification by Real-time RT-PCR assay for DENV, CHIKV, and ZIKV was performed with the primers and the probe used for DENV, CHIKV, and ZIKV described by Leparc-Goffart et al. [18], Palomares-Reyes [19], Panning M et al. [20] and Faye et al.[21], respectively. The PCR condictions were described by Sánchez-Carbonel et al. [22].

#### Real-time PCR assay for *Bartonella bacilliformis, Leptospira* spp. and *Rickettsia* spp*.*

The PCR was performed using primers and probe for species-specific gene of *Bartonella bacilliformis *[23], the PanR8 gene of *Rickettsia* spp.[24] and and the LipL32 gene of *Leptospira* spp.[25] as previously described. The PCR condictions were described by Ricapa-Antay et al., 2018 [15].

#### Data analysis

Qualitative variables were reported as frequencies and percentages. All analyses were processed with the IBM Statistical Package for the Social Sciences (SPSS) software version 21.0 (SPSS, Chicago, IL, USA).

### Results

A total of 124 blood samples from patients who presented with acute febrile illness were analyzed via PCR for laboratory identification of common etiologies included in the differential diagnosis.

DENV was the most common etiology detected in 25.8% of samples (32/124), followed by *Rickettsia* spp. in 8.1% (10/124), and ZIKV in 4.8% (6/124). Additionally, 3 samples were positive for CHIKV and 2 cases for *Bartonella* spp. There were no cases of OROV or *Lepstospira* spp. identified (Table 1). Interestingly, after PCR detection of the three-common sequence for all dengue viruses in our samples, we were able to identify serotypes in only 18.75% of samples (6/32). Using the standardized system proposed by Leparc-Goffart et al., we were only able to typify 2 samples for DENV-2 and 4 samples for DENV-4. Coinfection was observed only in 5 cases. Two samples were positive for DENV—*Rickettsia* spp, and one case of coinfections between CHIKV – *Rickettsia* spp., DENV – CHIKV and DENV – ZIKV were also observed.

**Table 1:** Demographics in patients with arboviral acute febrile illness

Age (years)	Total (%)	Real-time RT-PCR assay					Real-time PCR assay	
		DENV-2	DENV-3	Non-typeable DENV	Zika	Chikungunya	Bartonella	Rickettsia
	n = 124	n = 2	n = 4	n = 26	n = 6	n = 3	n = 2	n = 10
< 5	2 (1.6)	0 (0.0)	0 (0.0)	0 (0.0)	0 (0.0)	0 (0.0)	0 (0.0)	0 (0.0)
5–111	1 (0.8)	0 (0.0)	0 (0.0)	0 (0.0)	0 (0.0)	0 (0.0)	0 (0.0)	0 (0.0)
12–17	7 (5.6)	0 (0.0)	0 (0.0)	1 (3.8)	0 (0.0)	0 (0.0)	1 (50.0)	0 (0.0)
18–39	54 (43.6)	0 (0.0)	2 (50.0)	12 (46.1)	3 (50.0)	1 (33.3)	1 (50.0)	4 (40.0)
40–59	37 (29.8)	1 (50.0)	2 (50.0)	9 (34.6)	2 (33.3)	1 (33.3)	0 (0.0)	3 (30.0)
> 60	23 (18.5)	1 (50.0)	0 (0.0)	4 (15.4)	1 (16.7)	1 (33.3)	0 (0.0)	3 (30.0)
Gender								
Male	50 (40.3)	1 (50.0)	2 (50.0)	19 (73.0)	2 (33.3)	0 (0.0)	0 (0.0)	4 (40.0)
Female	74 (59.7)	1 (50.0)	2 (50.0)	7 (27.0)	4 (66.7)	3 (100.0)	2 (100.0)	6 (60.0)

The most common associated symptoms with fever were headache in 42.7% (53/124), followed by joint pain in 27.4% (34/124), and muscle pain in 23.4% (29/124). Additionally, headache was reported as the most common symptom across all etiologies. No symptom predominance was observed for any particular infective agent. (Table 2).

**Table 2:** Clinical symptoms in patients with arbovirus infection confirmed by PCR

Clinical symptoms	Total n = 124 (%)	Dengue	Zika	Chikungunya	Bartonella	Rickettsia
		n = 32 (%)	n = 6 (%)	n = 3 (%)	n = 2 (%)	n = 10 (%)
Chills	10 (8.06)	1 (3.13)	0 (0.00)	0 (0.00)	1 (50.00)	0 (0.00)
Headache	53 (42.74)	13 (40.63)	2 (33.33)	2 (66.67)	2 (100.00)	3 (30.00)
Dizziness	3 (2.42)	0 (0.00)	0 (0.00)	0 (0.00)	2 (100.00)	0 (0.00)
Rhinorrhea	1 (0.81)	0 (0.00)	0 (0.00)	0 (0.00)	0 (0.00)	0 (0.00)
Cough	1 (0.81)	0 (0.00)	0 (0.00)	0 (0.00)	0 (0.00)	0 (0.00)
Shortness of breath	2 (1.62)	0 (0.00)	0 (0.00)	0 (0.00)	1 (50.00)	0 (0.00)
Nausea/vomiting	14 (11.29)	3 (9.38)	0 (0.00)	0 (0.00)	1 (50.00)	0 (0.00)
Abdominal pain	8 (6.45)	1 (3.13)	0 (0.00)	0 (0.00)	1 (50.00)	0 (0.00)
Diarrhea	7 (5.65)	1 (3.13)	0 (0.00)	0 (0.00)	1 (50.00)	0 (0.00)
Myalgias	29 (23.39)	7 (21.88)	0 (0.00)	0 (0.00)	1 (50.00)	4 (40.00)
Arthralgias	34 (27.42)	6 (18.75)	1 (16.67)	2 (66.67)	0 (0.00)	0 (0.00)
Conjunctivitis	2 (1.61)	0 (0.00)	0 (0.00)	0 (0.00)	0 (0.00)	0 (0.00)
Retroocular pain	23 (18.55)	7 (21.88)	1 (16.67)	0 (0.00)	0 (0.00)	1 (10.00)
Lumbar pain	14 (11.29)	3 (9.38)	1 (16.67)	0 (0.00)	0 (0.00)	0 (0.00)
Rash	10 (8.06)	3 (9.38)	0 (0.00)	0 (0.00)	0 (0.00)	1 (10.00)

**Fig. 1:**
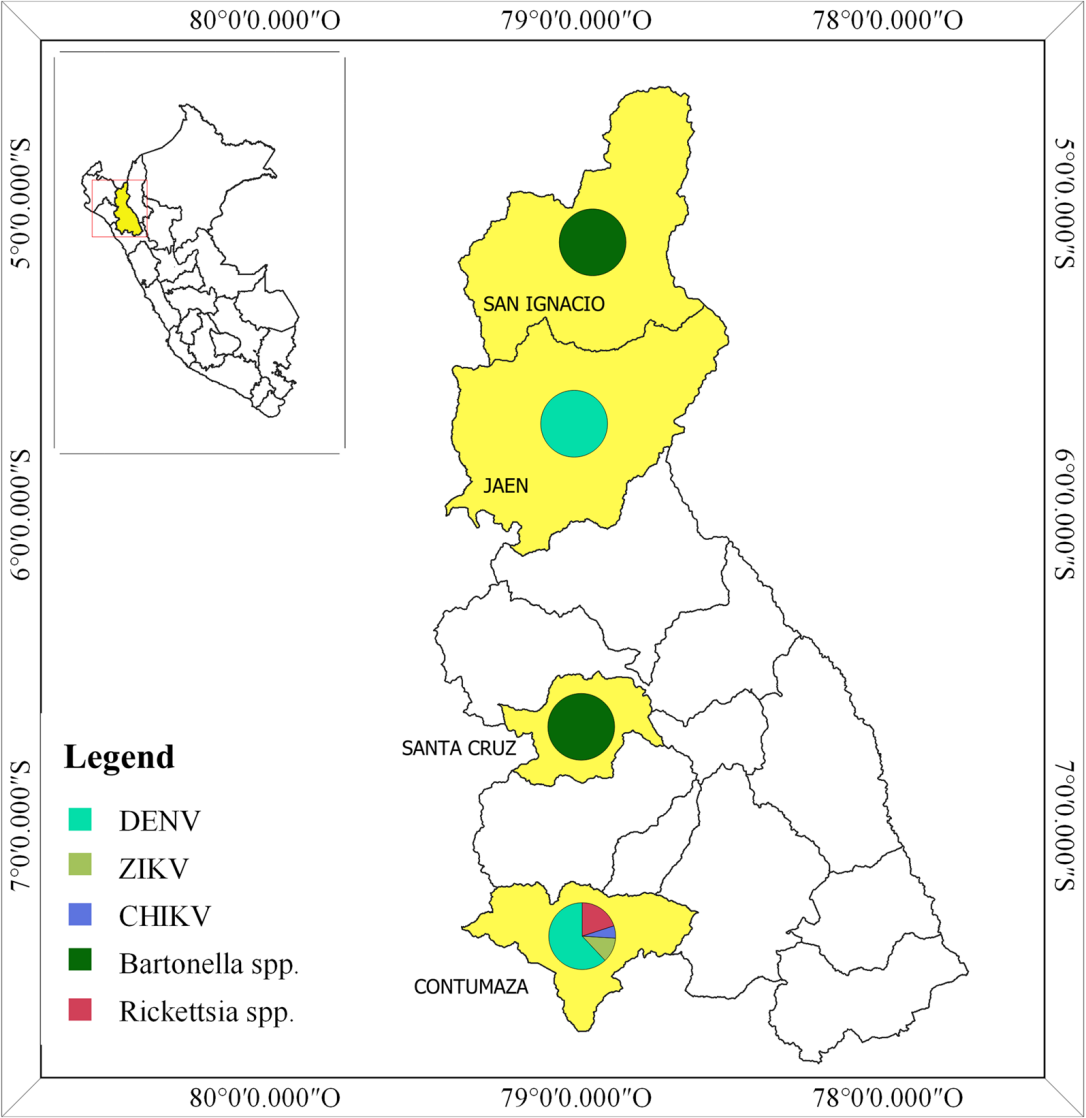
Arbovirus infection geographic distribution in Cajamarca between February to June 2016

A geographic map was performed based on the distribution of the isolated pathogens. Almost all cases of DENV (31/32) were isolated on Contumaza city located at the south of Cajamarca. In the same city, 10 cases of *Rickettsia*, 6 cases of ZIKV and 3 cases of CHIKV were found. Only 1 case of DENV was isolated in Jaen, located on the northern Cajamarca. The 2 cases of *Bartonella* were isolated on San Ignacio city and Santa Cruz city (Fig. 1).

### Discussion

In recent years, important advances have been made that reduce, but not eliminate, uncertainty in diagnosing the cause of acute febrile illness in the context of medical practice with limited resources [1]. This fact has allowed to detect some emerging infectious diseases early and identify pathogens that were believed to be eradicated in other contexts. As is the case of arboviruses, involved in the increase in morbidity and mortality due to infectious causes in Latin America, which urges to improve their epidemiological surveillance [7, 8, 9, 26]. Currently, in an age of constantly emerging and re-emerging pathogens, knowing the etiology of AFI would allow targeted treatment, rational use of antibiotics and improvement in patient care in resource-limited settings [1, 2]. For this reason, this study aimed to describe the prevalence of pathogens responsible for an AFI outbreak 2016 in the region of Cajamarca, Peru.

In our study, DENV was identified as the most frequent etiologic agent responsible for AFI in 25.8% (32/124) of the samples, followed by *Rickettsia* spp. in 8.1% (10/124) and ZIKV in 4.8% (6/124). However, we could only determine the infecting DENV serotype in 18.8% (6/32) of the samples, with two positive samples for DENV-2 and four positive samples for DENV-4, in the remaining 81.2% (26/32) it was not possible to determine the viral serotype. The low frequency of serotype identification could be explained by the following reasons.

First of all, DENV possesses a positive-sense single-stranded RNA genome with an RNA-dependent RNA polymerase lacking corrective activity, which can result in a mutation for each round of replication of its genome [10, 26, 27]. The accumulation of these mutations in their genome can alter the hybridization of oligonucleotides with the target sequences of the circulating strains generating false negatives [28].

Another possibility is that our samples do not correspond to any of the DENV 1–4 serotypes. These serotypes evolved independently from their respective DENV sylvatic progenitor and presented an amino acid divergence of 30–35% with a genotypic classification defined as arbitrary [29, 30]. Recently, the appearance of a fifth DENV serotype has been reported, which possess a different viral sequence than previous sylvatic and human DENV strains. Moreover it induces a different antibody response than the DENV 1- 4 serotypes [31]. It is believed that there is a continuous propagation of sylvatic DENV in humans, which can cause sporadic infections without entering an epidemic-urban cycle and that there is a considerable diversity of unspecified jungle DENV [29, 32, 33]. Either scenario demonstrates the rapid diversification of the Dengue virus and understanding its evolution allows us to improve our surveillance systems and the response to epidemics.

Less frequently, but no less important epidemiologically, we found pathogens such as *Rickettsia* spp., *Bartonella* spp., ZIKV and CHIKV. This study reports the first cases of CHIKV in Cajamarca demonstrating its potential as an emerging virus. The transmission is likely to come from the coastal regions of northern Peru, with a tendency to cover new geographical territories [22]. Regarding Zika virus, we can highlight its presence in in an area where Dengue is currently circulating. Current studies seek to determine if cross-immunity can give relative protection against ZIKV in human hosts, which could explain the few registered cases [34]. On the other hand, cases of Ricketssia were found, which is described as an emerging pathogen, perhaps due to a growing interest in its identification [17].

Regarding clinical symptoms evaluated in our study, we found that there were no clear differences in the clinical presentation between the pathogens, This highlights the similarity in the clinical picture of these infective agents. As mentioned before, accurate diagnostic laboratory methods are required to decrease the uncertainty in the diagnosis of current emerging and reemerging causes of AFI.

In conclusion, during the 2016 outbreak in Cajamarca, Peru, it was observed that in a significant percentage of positive samples for DENV it was not possible to determine the serotype by using conventional detection assays, such as that validated by Leparc-Goffart et al. in 2009. This represents a problem for the national surveillance system and for public health due to its epidemiological and clinical implications. Other viral and bacterial pathogens responsible for acute febrile syndrome were identified less frequently, among them we highlight the detection of CHIKV in this region for the first time.

## Limitations

The main limitation of this study was the fact that the research team could not perform further viral phylogenetic analyzes in order to explain, deepen or expand our results. This due to limited funding for this project. However, all samples are stored for possible phylogenetic classification in the future as it is a priority within our line of research. Given our limited serotyping rate, further analysis of the clinical presentation was not possible.

## Data Availability

Abstraction format used in the study and dataset are available and accessible from the corresponding author upon request in the link: https://figshare.com/s/8b711e013e90a5780ab6

## Abbreviations

DENV: Dengue
CHIKV: Chikungunya
ZIKV: Zika
OROV: Oropouche
RT-PCR: Reverse transcription polymerase chain reaction
PCR: Reaccion polimerasa chain
DNA: Deoxyribonucleic acid
RNA: Ribonucleic acid; bp: base pairs
